# An Explanation

**Published:** 1893-07

**Authors:** 


### AN EXPLANATION.

In the July number of this journal for 1890, pages 402 and 403, appears a letter signed "A Patient," and on page 705 of the November number (1890) a communication to the editor by the same person. The second paragraph of the latter would appear to cast the reflection upon the author of the paper, to which the letter is appended in the July number, that he had made an unauthorized use of this letter.

The present editor of the Journal, in a spirit of fairness, permitted the second communication to appear (at the request of the author of both letters), with the belief that further explanation by the parties would bring out the facts. To his surprise, this was not done, so far as this journal is concerned.

The case was, however, brought before the Council of the First District Society of New York, and it was made manifest to that body that the letter published on pages 402 and 403 of 1890, before alluded to, was a genuine one, and that the patient had authorized its use, on condition that his proper name be protected from publicity. These pages would have been open at the time for the statement of the above facts or any others which would have justly protected either of the parties. We can only regret at this late date that the Journal was not given the opportunity to know the facts, and thereby relieve Dr. Kasson C. Gibson from the imputation of want of sincerity in using the letter of his patient.

